# Supplementary material for: Longevity in C. elegans Eat mutants is largely attributable to reduced bacterial infection
Source: bioRxiv. 2026 Jul 13:2026.03.11.711062. Preprint. [Version 2] doi: 10.64898/2026.03.11.711062 (PMC13404839; doi:10.64898/2026.03.11.711062)
Supplement: Supplement 2 [file NIHPP2026.03.11.711062v2-supplement-2.pdf]

bioRxiv preprint doi: https://doi.org/10.64898/2026.03.11.711062; this version posted July 13, 2026. The copyright holder for this preprint (which was not certified by peer review) is the author/funder, who has granted bioRxiv a license to display the preprint in perpetuity. It is made available under a CC-BY 4.0 International license.

Supplementary Information

Longevity in *C. elegans* Eat mutants is largely attributable to reduced bacterial infection

Contents Summary

Supplementary Figure 1. Mortality deconvolution data for selected Eat mutants.

Supplementary Figure 2. Site of initiation of infection in the pharyngeal bulb anterior.

Supplementary Figure 3. Carbenicillin has little effect on reduced reproduction in Eat mutants.

Supplementary Figure 4. Effects of 3 *eat-2* alleles on overall and p subpopulation lifespan, and P death frequency.

Supplementary Figure 5. Correlation between metrics of malnutrition and early bacterial invasion in Eat mutants.

Supplementary Figure 6. Correlation between mean lifespan in carbenicillin treated worms with metrics of malnutrition.

Supplementary Figure 7. Regression analysis of lawn avoidance.

Supplementary Figure 8. Effects of carbenicillin on *phm-2(ad597)* mutants on large lawns.

Supplementary Table 1. Previous findings employing *eat-2* mutants as a model for dietary restriction.

Supplementary Table 2. Lifespan analysis of Eat mutants on proliferating bacteria (20˚C).

Supplementary Table 3. Lifespan analysis of Eat mutants on non-proliferating bacteria (20˚C).

Supplementary Table 4. Lifespan analysis of 3 *eat-2* mutants on proliferating bacteria (20˚C).

Supplementary Table 5. Lifespan analysis of *eat-2* and *phm-2* mutants on small and large bacterial lawns (20˚C).

bioRxiv preprint doi: https://doi.org/10.64898/2026.03.11.711062; this version posted July 13, 2026. The copyright holder for this preprint (which was not certified by peer review) is the author/funder, who has granted bioRxiv a license to display the preprint in perpetuity. It is made available under a CC-BY 4.0 International license.

Supplementary figures

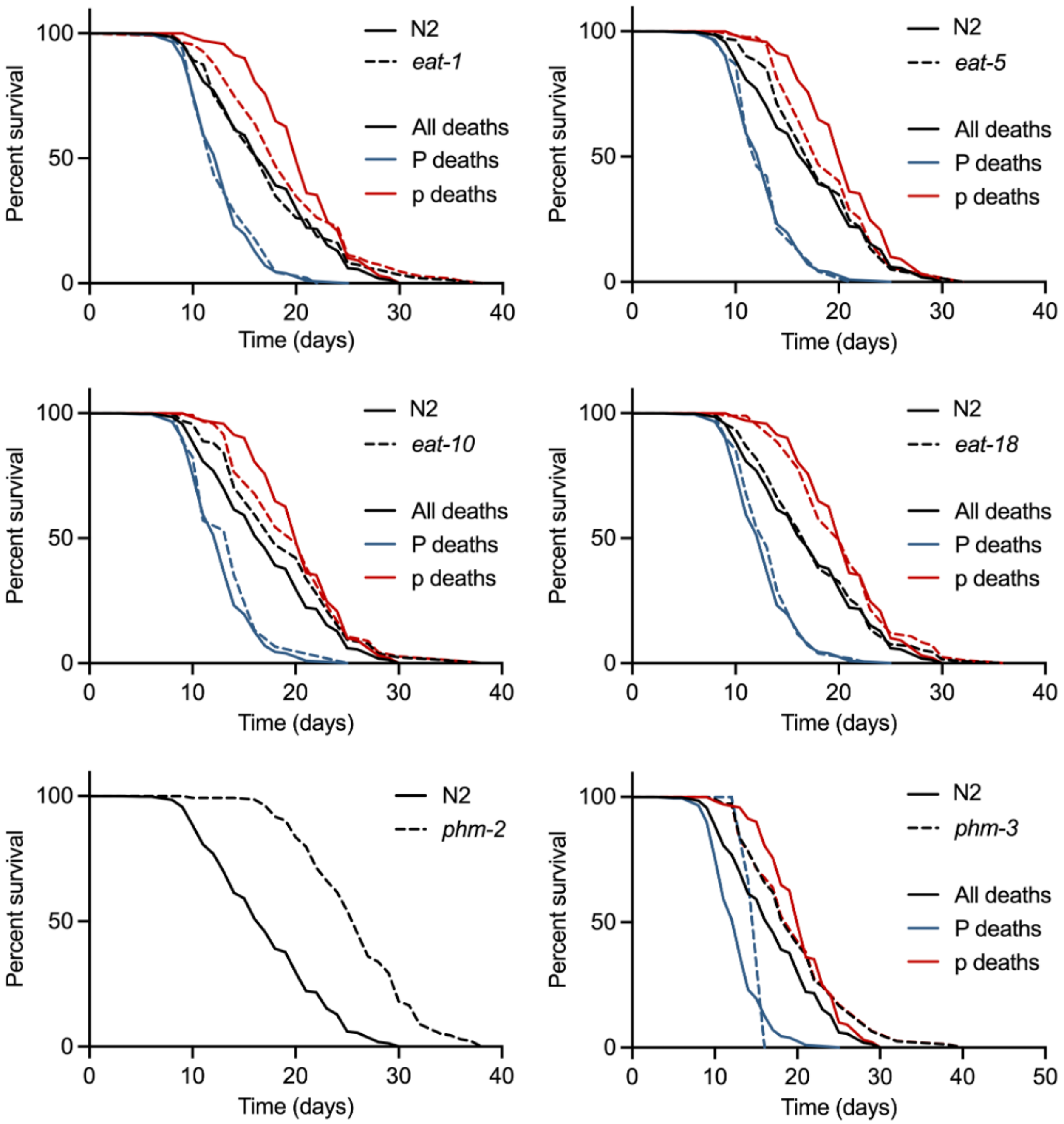

Supplementary Figure 1. Mortality deconvolution data for selected Eat mutants. Survival curves of the whole population, the P sub-population and the p sub-population.

bioRxiv preprint doi: https://doi.org/10.64898/2026.03.11.711062; this version posted July 13, 2026. The copyright holder for this preprint (which was not certified by peer review) is the author/funder, who has granted bioRxiv a license to display the preprint in perpetuity. It is made available under a CC-BY 4.0 International license.

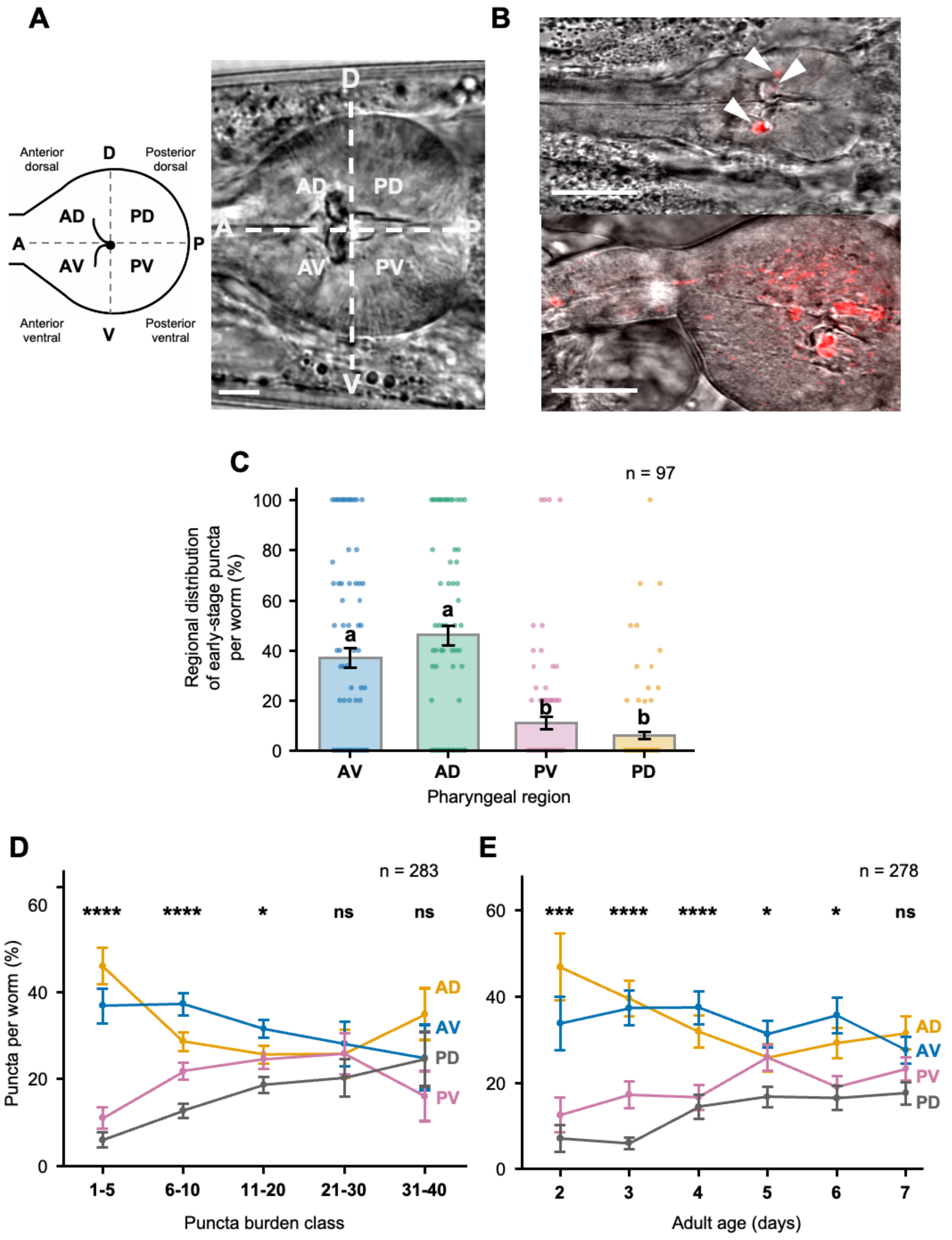

Supplementary Figure 2. Site of initiation of infection in the pharyngeal bulb anterior. (**A**) Four quadrants of the terminal pharyngeal bulb: AV, anterior ventral, AD, anterior dorsal, PV, posterior ventral, PD, posterior dorsal. Left, schematic representation of the pharyngeal bulb. Right, brightfield image (Nomarski). Scale bar: 10 µM. (**B**) Example of pharyngeal infection with *E. coli* OP50 expressing RFP. Top, early stage infection (day 5 of adulthood). Arrowheads: localized infection visible as red fluorescent puncta in anterior dorsal and anterior ventral quadrants (2 and 1 puncta/punctum, respectively). Bottom, example of advanced stage infection (day 9 of adulthood, live individual), where numerous individual puncta have coalesced, such that counting them is no longer practicable. Note the swollen state of the pharynx, preceding P death. Merged bright field and epifluorescence images. Scale bar: 20 µM. (**C**) Early-stage infection (1–5 discrete RFP puncta) in the four pharyngeal quadrants, showing elevated frequency in anterior quadrants (n = 97 animals, mainly D2-D4 of adulthood). (**D**) Puncta number differs between pharyngeal quadrants only at lower puncta frequencies (mostly D1-D7 animals). (**E**) Puncta number differs between pharyngeal quadrants only at earlier ages. Individuals with swollen, pre-P death pharynxes were not included in this analysis, due to puncta no longer being countable. (**C**-**E**) ns $p > 0.05$, * $p \leq 0.05$, ** $p \leq 0.01$, *** $p \leq 0.001$, **** $p \leq 0.0001$, pairwise comparisons using Holm-corrected Wilcoxon signed-rank test.

bioRxiv preprint doi: https://doi.org/10.64898/2026.03.11.711062; this version posted July 13, 2026. The copyright holder for this preprint (which was not certified by peer review) is the author/funder, who has granted bioRxiv a license to display the preprint in perpetuity. It is made available under a CC-BY 4.0 International license.

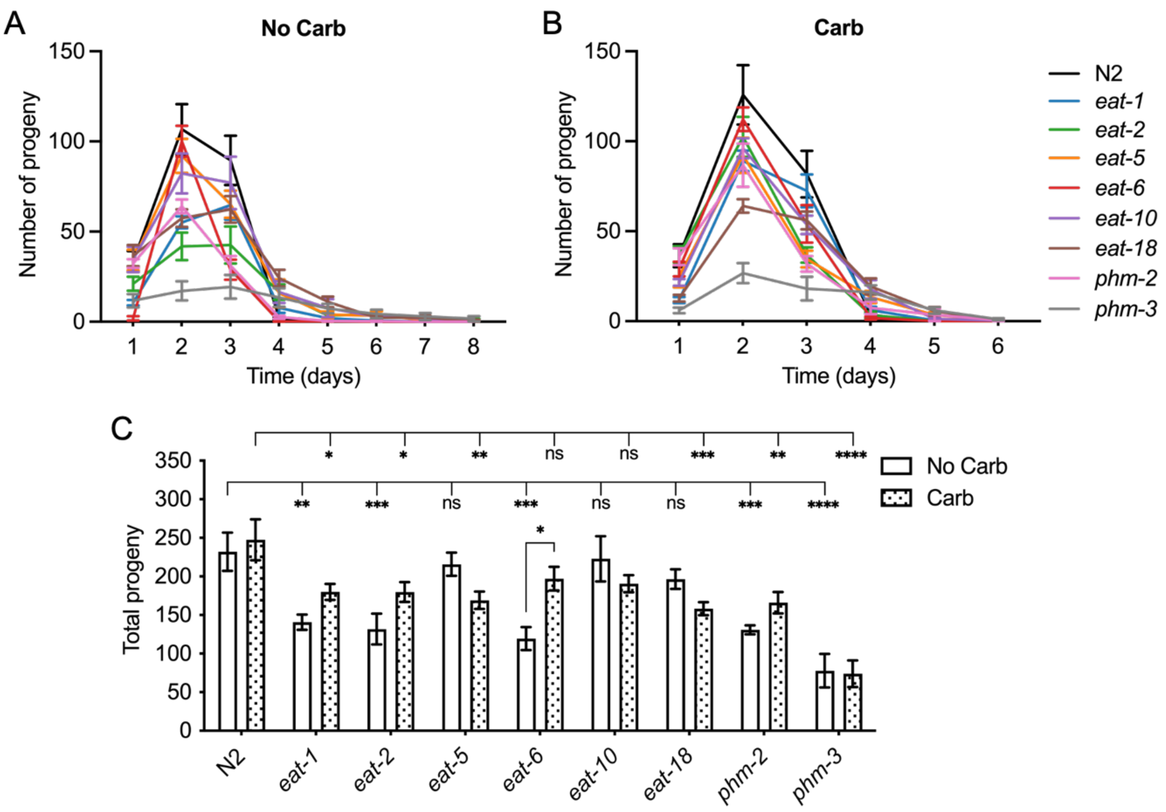

Supplementary Figure 3. Carbenicillin has little effect on reduced reproduction in Eat mutants. (**A, B**) Reproductive schedule, no Carb (**A**), Carb (**B**). (**C**) Brood size. Dunnett's multiple comparisons test, *p<0.05, **p<0.01, ***p<0.001, ****p<0.0001. n=7-10 for each strain.

bioRxiv preprint doi: https://doi.org/10.64898/2026.03.11.711062; this version posted July 13, 2026. The copyright holder for this preprint (which was not certified by peer review) is the author/funder, who has granted bioRxiv a license to display the preprint in perpetuity. It is made available under a CC-BY 4.0 International license.

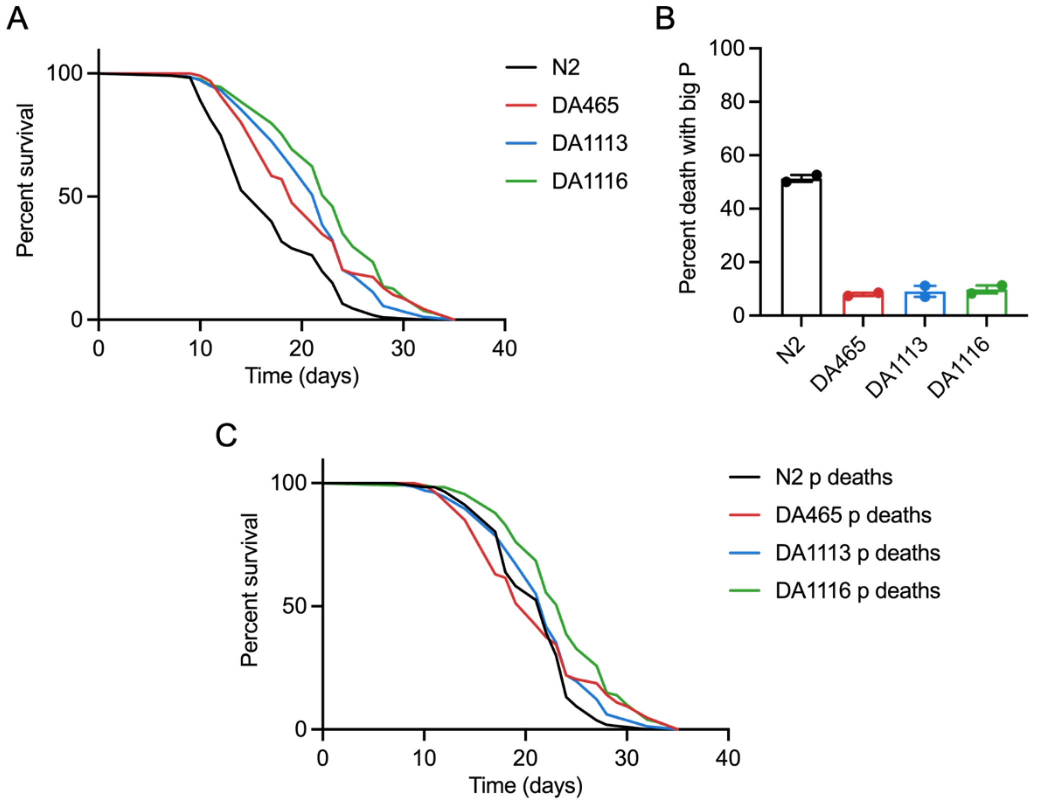

Supplementary Figure 4. Effects of 3 *eat-2* alleles on overall and p subpopulation lifespan, and P death frequency. Culture on proliferating *E. coli* OP50. (**A**) Lifespans of overall populations, pooled data. (**B**) P death frequency, pooled data. (**C**) Lifespans of p subpopulations, pooled data. $N = 2$.

bioRxiv preprint doi: https://doi.org/10.64898/2026.03.11.711062; this version posted July 13, 2026. The copyright holder for this preprint (which was not certified by peer review) is the author/funder, who has granted bioRxiv a license to display the preprint in perpetuity. It is made available under a CC-BY 4.0 International license.

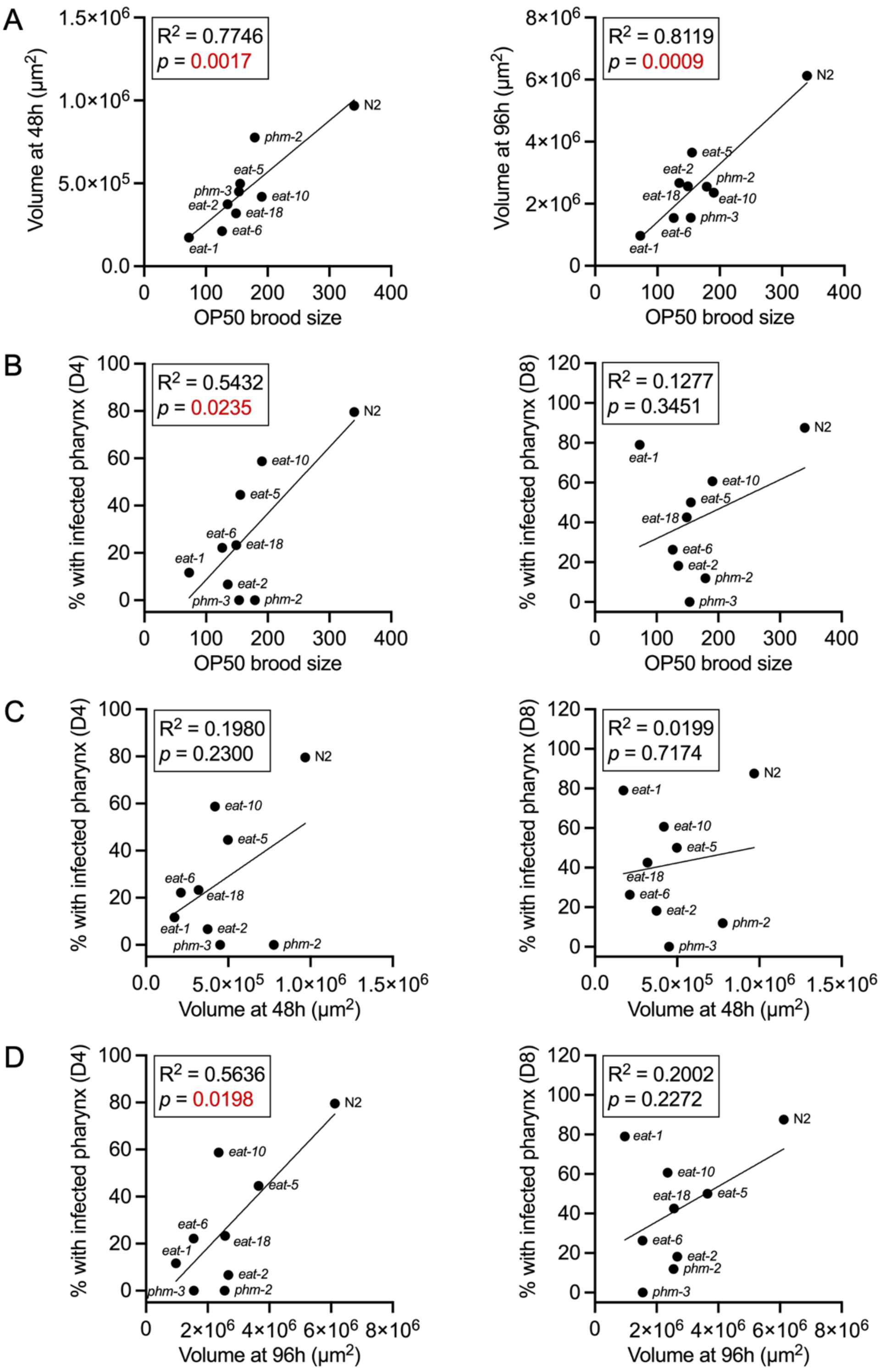

Supplementary Figure 5. Correlation between metrics of malnutrition and early bacterial invasion in Eat mutants. (**A**) Correlations between body size (estimated volume) and brood size. (**B**) Correlation between brood size and either early bacterial infection on day 4 (left) or day 8 (right). (**C**) Correlation between 48h body size and either early bacterial invasion on day 4 (left) or day 8 (right). (**D**) Correlation between 96h body size and either early bacterial invasion on day 4 (left) or day 8 (right).

bioRxiv preprint doi: https://doi.org/10.64898/2026.03.11.711062; this version posted July 13, 2026. The copyright holder for this preprint (which was not certified by peer review) is the author/funder, who has granted bioRxiv a license to display the preprint in perpetuity. It is made available under a CC-BY 4.0 International license.

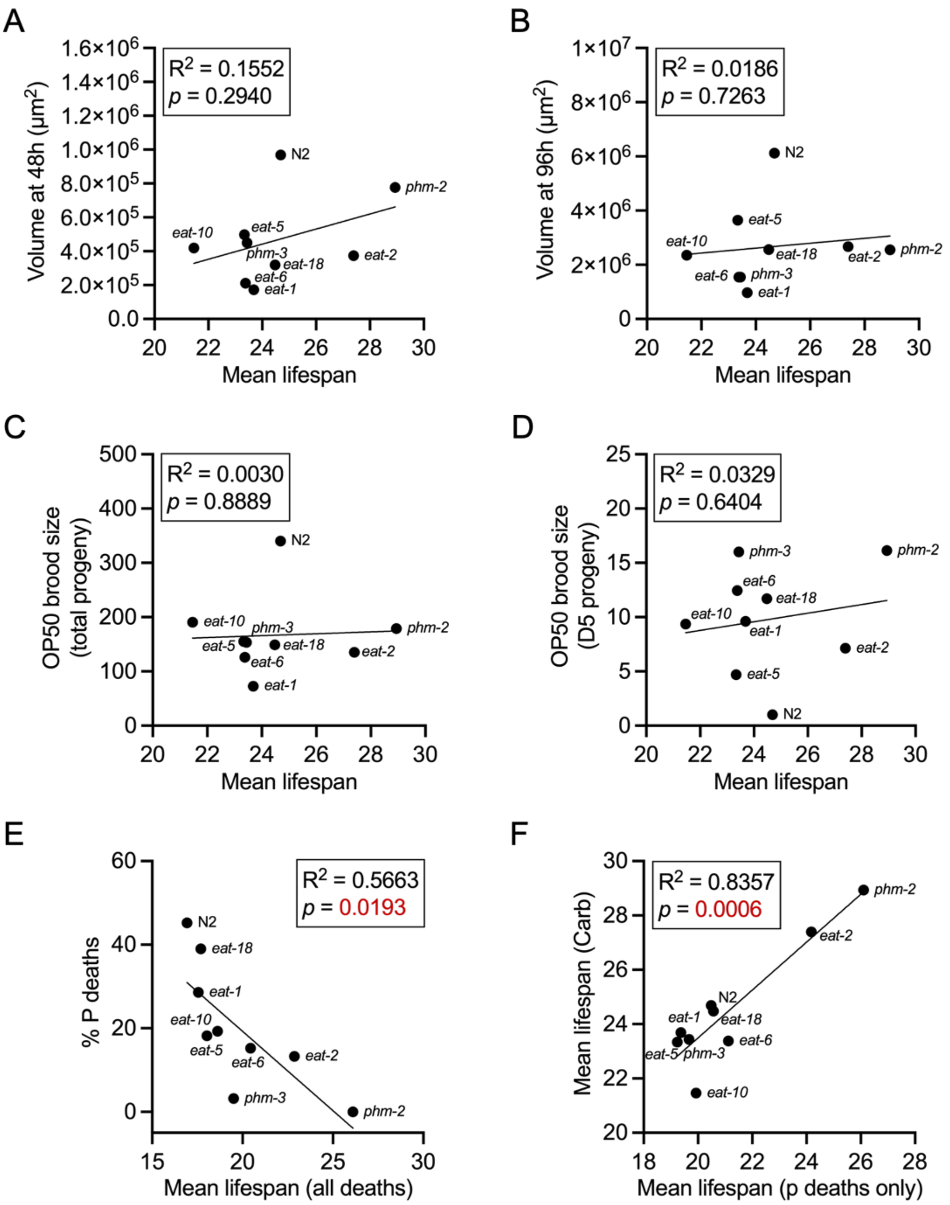

Supplementary Figure 6. Correlation between mean lifespan in carbenicillin-treated worms with metrics of malnutrition. (**A**) Correlation with body size (estimated volume) at 48h after egg lay. (**B**) Correlation with body size (estimated volume) at 96h after egg lay. (**C**) Correlation with brood size. (**D**) Correlation with Day 5 progeny count. (**E**) Correlation between overall lifespan with % P deaths. (**F**) Correlation of lifespan between p populations (no carbenicillin) and carbenicillin-treated populations.

bioRxiv preprint doi: https://doi.org/10.64898/2026.03.11.711062; this version posted July 13, 2026. The copyright holder for this preprint (which was not certified by peer review) is the author/funder, who has granted bioRxiv a license to display the preprint in perpetuity. It is made available under a CC-BY 4.0 International license.

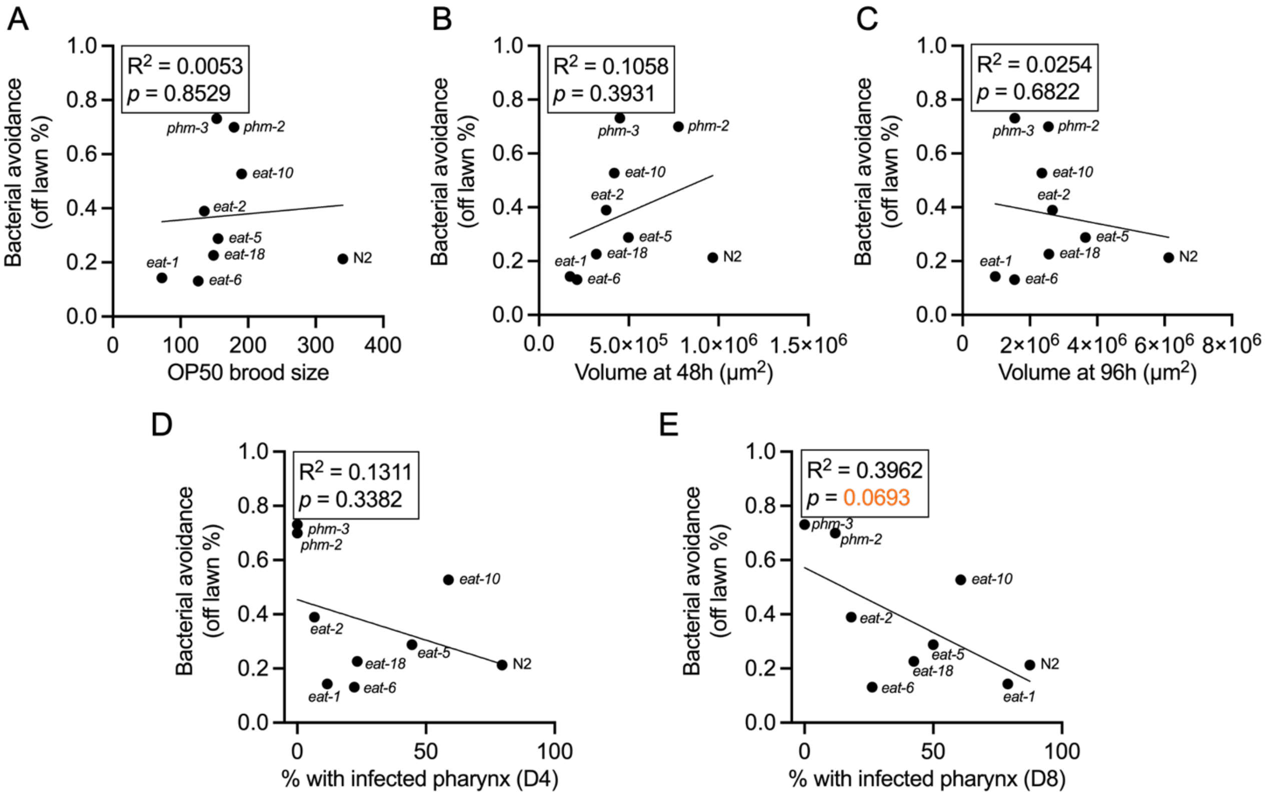

Supplementary Figure 7. Regression analysis of lawn avoidance. (**A**) Correlation with brood size. (**B**) Correlation with body size (estimated volume) at 48h after egg lay. (**C**) Correlation with body size (estimated volume) at 96h after egg lay. (**D, E**) Correlation with bacterial invasion on day 4 (**D**) and day 8 (**E**).

bioRxiv preprint doi: https://doi.org/10.64898/2026.03.11.711062; this version posted July 13, 2026. The copyright holder for this preprint (which was not certified by peer review) is the author/funder, who has granted bioRxiv a license to display the preprint in perpetuity. It is made available under a CC-BY 4.0 International license.

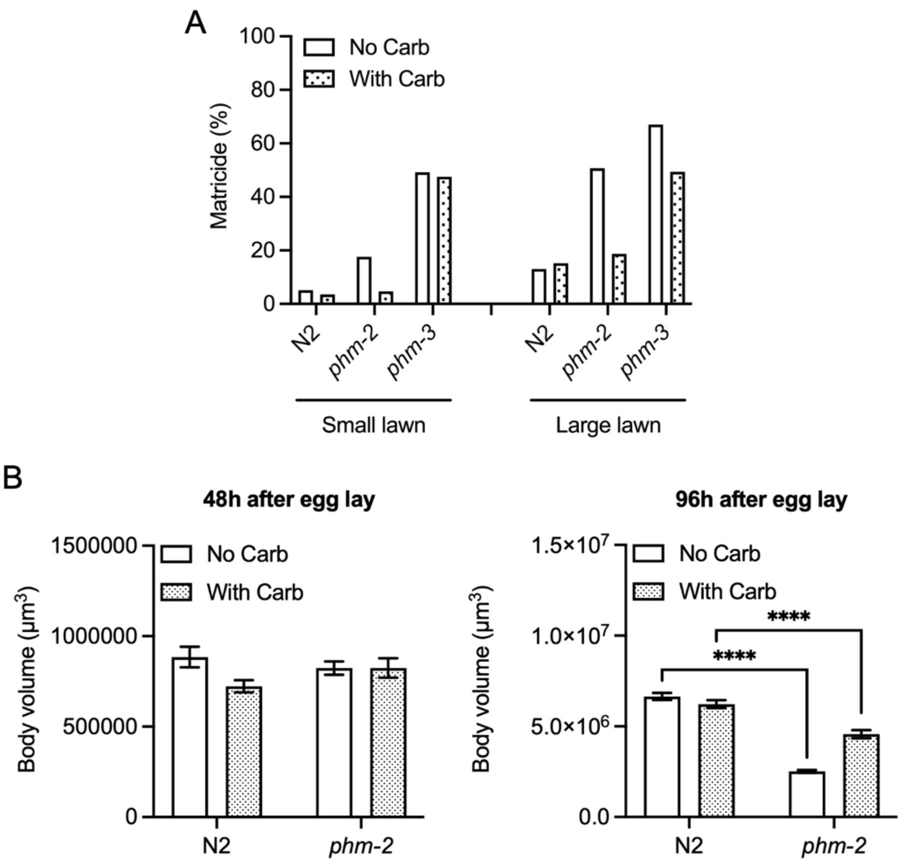

Supplementary Figure 8. Effects of carbenicillin on *phm-2(ad597)* mutants on large lawns. (**A**) Carbenicillin suppresses elevated levels of matricide in *phm-2* mutants on large lawns. (**B**) Carbenicillin rescues the reduction in body size at 96 hr in *phm-2* mutants on large lawns. Thus, on large lawns proliferative *E. coli* causes matricide and reduces growth in *phm-2* mutants.

bioRxiv preprint doi: https://doi.org/10.64898/2026.03.11.711062; this version posted July 13, 2026. The copyright holder for this preprint (which was not certified by peer review) is the author/funder, who has granted bioRxiv a license to display the preprint in perpetuity. It is made available under a CC-BY 4.0 International license.

Supplementary Table 1. Findings from 77 previous studies employing *eat-2* mutants as a model for dietary restriction

**Authors**	**Key findings relating to *eat-2***
(Lakowski and Hekimi, 1998)	Large lifespan extensions for *eat-2*, for all 4 alleles tested (Fig. 1, Table 1). *eat-2(ad465)* longevity is not suppressed by *daf-16(m26)* (Fig. 2B). Effects of *eat-2(ad465)* and *daf-2(e1370)* on longevity are approximately additive (Fig. 3A).
(Hsu et al., 2003)	Effects of *eat-2(ad1116)* and *hsf-1* RNAi (heat shock factor 1) on longevity are approximately additive (Fig. 1C,D), i.e. *hsf-1* RNAi does not suppress *eat-2* longevity.
(Huang et al., 2004)	*eat-2(ad465)* extends reproductive span, fast body movement span, pharyngeal pumping span (Fig. 2, Table 1).
(Curtis et al., 2006)	Neither *eat-2(ad1116)* or *eat-2(ad465)* longevity are suppressed by *aak-2(ok524)* (AMP kinase) (Table 2).
(Pinkston et al., 2006)	*eat-2(ad1116)* extends the lifespan of *gld-1* RNAi-treated (defective in Germ Line Development) animals that are short-lived due to germline tumors (Fig. 1E).
(Wang and Tissenbaum, 2006)	*sir-2.1(ok434)* (sirtuin) largely and fully suppresses *eat-2(ad465)* and *eat-2(ad1113)* longevity, respectively (Fig. 3C,D).
(Crawford et al., 2007)	Effects of *eat-2(ad1116)* and germline ablation on longevity are approximately additive (Fig. 2C, Table 1).
(Hansen et al., 2007)	*sir-2.1(ok434)* (sirtuin) does not suppress *eat-2(ad1116)* longevity (Fig. 5, Table S4).
(Iser and Wolkow, 2007)	*eat-2(ad465)* induces a FIRE (Fasting-Induced Redistribution of Esterase activity) response (Fig. 1B) and further increases *daf-2(e1370)* lifespan (Fig. 5).
(Jia and Levine, 2007)	*bec-1* RNAi and *atg-7* RNAi (autophagy) suppress *eat-2(ad1116)* longevity (Fig. 1, Table 1).
(Murakami and Murakami, 2007)	Effects of *eat-2(ad465)* and *ser-1* RNAi (serotonin receptor) on longevity are approximately additive (Fig. 2A, Table 2).
(Pan et al., 2007)	Effects of *eat-2(ad465)* and *rsks-1*(*ok1255*) (S6 kinase) or *ifg-1* RNAi (eIF4G) on longevity are approximately additive (Fig. 2G).
(Panowski et al., 2007)	*smk-1* or *pha-4* RNAi suppress *eat-2(ad1116)* longevity (Fig. 1A,C).
(Petrascheck et al., 2007)	*eat-2(ad1116)* suppresses life extension by mianserin (antidepressant drug) (Fig. 3B, Table 1).
(Syntichaki et al., 2007)	Effects of *eat-2(ad465)* and *ife-2* RNAi (translation initiation factor eIF4E) on longevity are approximately additive (Fig. 2E, Table S1).
(Galbadage and Hartman, 2008)	Effects of *eat-2(ad465)* and thermocycling between 12˚C and 25˚C at 10-min intervals on longevity are approximately additive (Fig. 2B, Table 1).
(Hansen et al., 2008)	*bec-1* and *vps-34* (autophagy) RNAi suppress *eat-2(ad1116)* longevity (Fig. 2A-D, Table 1).
(Toth et al., 2008)	*bec-1(ok691)* and *unc-51(e1189)* (autophagy) suppress *eat-2(ad1116)* longevity (Fig. 7).
(Artal-Sanz and Tavernarakis, 2009)	Effects of *eat-2(ad465)* and *phb-1* or *phb-2* RNAi (prohibitin) on longevity are approximately additive (Fig. 2E, Table S1).
(Carrano et al., 2009)	*wwp-1* RNAi (ubiquitin ligase) suppresses *eat-2(ad1116)* longevity (Fig. 1B, Table S1).
(Chen et al., 2009)	*egl-9(sa307)* (increases HIF-1 [hypoxia inducible factor-1] activity) suppresses *eat-2(ad1116)* longevity (Fig. 4, Table 2, Table S2).
(Greer and Brunet, 2009)	*daf-16(mu86)* (FOXO transcription factor) and *aak-2(ok524)* (AMP kinase) do not suppress *eat-2(ad1116)* longevity (Fig. 2C,D, Table S4)
(Mehta et al., 2009)	*hif-1* RNAi (hypoxia-inducible factor) does not suppress *eat-2(ad465)* longevity (Fig. 3).
(Schlotterer et al., 2009)	*eat-2(ad465)* lifespan is reduced by high glucose treatment (Table 1).
(Van Raamsdonk and Hekimi, 2009)	Life-extending effects of *eat-2(ad1116)* and *sod-2(ok1030)* (deletion allele) have only a very weak additive effect (Fig. 5D).
(Ching et al., 2010)	Over-expression of *drr-2* (translation initiation factor eIF4H) largely suppresses *eat-2(ad1116)* longevity (Fig. 3B, Table S1).
(Lee et al., 2010)	*hif-1* RNAi (hypoxia-inducible factor) does not suppress *eat-2(ad1116)* longevity (Fig. S2I, S2J).
(Onken and Driscoll, 2010)	Metformin (anti-diabetic drug) extends lifespan in N2 but not *eat-2(ad1116)* mutants (Fig. 2A, Table S1D).
(Park et al., 2010)	*eat-2(ad465)* lifespan is shortened by RNAi of *skn-1* (NRF-2-like transcription factor), *nlp-7* (neuropeptide) and *cup-4* (coelomocyte ion channel) (Fig 1A, Table S2).

bioRxiv preprint doi: https://doi.org/10.64898/2026.03.11.711062; this version posted July 13, 2026. The copyright holder for this preprint (which was not certified by peer review) is the author/funder, who has granted bioRxiv a license to display the preprint in perpetuity. It is made available under a CC-BY 4.0 International license.

(Powolny et al., 2011)	10 μM diallyl trisulfide (garlic constituent) extends N2 but not *eat-2(ad1113)* lifespan (Fig. 3A, Table 1).
(Yuan et al., 2012)	*sbds-1* RNAi (ribosome maturation factor) slightly reduces and *hpd-1* RNAi (4-hydroxyphenylpyruvate dioxygenase) slightly increases *eat-2(ad1116)* lifespan (Fig. 6, Table S2).
(Vilchez et al., 2012)	*rpn-6.1* RNAi (proteasome) suppresses *eat-2(ad1116)* longevity (Fig. S19E).
(Heestand et al., 2013)	*nhr-62(tm1818)* (HNF4α-related nuclear hormone receptor) suppresses *eat-2(ad465)* longevity (Fig 1F).
(Chamoli et al., 2014)	*mekk-3* RNAi (kinase) slightly reduces *eat-2(ad1116)* lifespan (Fig. 2B, Table 1).
(Yee et al., 2014)	*ced-4(n1162)* (apoptosis) had no effect on *eat-2(ad1116)* longevity (Fig. S4).
(Bansal et al., 2015)	*eat-2(ad1113)* extends lifespan but not healthspan, with respect to both chronological and biological age.
(Palikaras et al., 2015)	*dct-1* RNAi (mitophagy) weakly suppresses *eat-2(ad465)* longevity (Fig. S8H).
(Gelino et al., 2016)	Intestinal autophagy is increased in *eat-2(ad1116)*, which is required to maintain intestinal barrier integrity and motility during aging.
(Singh et al., 2016)	*pha-4* (FOXA transcription factor), *zfp-1* (chromatin modifier) and *gfl-1* (GAS41) RNAi all markedly reduce *eat-2*(*ad1116*) lifespan (Fig. 6D, Table S1, S3).
(Tabrez et al., 2017)	*smg-2* RNAi (nonsense-mediated decay, regulated by PHA-4/FOXA) suppresses *eat-2(ad1116)* longevity (Fig. 4C, Table S4). *eat-2* longevity may involve alternative splicing, nonsense-mediated decay.
(Chen et al., 2018)	Lifespan-extension by echinacoside (phenylethanoid glycoside from *C. deserticola*) is dependent on *eat-2(ad1116)* (Fig. 6A).
(Meng et al., 2018)	Gengnianchun (traditional Chinese medicine mixture) increases *eat-2(ad465)* lifespan (Fig. 7A).
(Mi et al., 2018)	Methyl 3,4-dihydroxybenzoate increases *eat-2(ad1113)* lifespan (Fig. 5).
(Yang et al., 2018)	*Lonicera japonica* extract increases *eat-2(ad1116)* lifespan (Fig. 14).
(Gao et al., 2018)	Common transcriptomic and metabolomic signatures between *eat-2(ad465)* and *daf-2(e1370)*, including increased amino acid metabolism and purine biosynthesis pathway.
(Admasu et al., 2018)	Complete lipid profile of *eat-2(ad1116)*.
(Kim et al., 2018)	Lifespan-extension by selenocysteine and *eat-2(ad465)* occur through shared pathways (Fig. 1, Table 1).
(Shpigel et al., 2019)	*eat-2(ad453)* and *eat-2(ad1116)* maintain heat shock response activation and protein folding capacity during early-mid adulthood.
(García-Casas et al., 2018)	Benzothiazepine CGP37157 increases *eat-2(ad1113)* lifespan (Fig. 2, Table 3).
(Zhu et al., 2019)	Aiweixin (traditional Uyghur medicine) increases *eat-2(ad465)* lifespan (Table 2).
(Kozlova et al., 2019)	Slow larval growth and long lifespan of *eat-2* mutants results from cross-talk with *gar-3* (a muscarinic ACh receptor).
(Kumar et al., 2019)	Intestinal *E. coli* colonization in *eat-2(ad1116)*, *eat-2(ad465)* and *phm-2* due to defective grinder function causes lawn avoidance and subsequent dietary restriction-mediated longevity.
(Hahm et al., 2019)	*zip-2* is activated in an *eat-2* mutant and is required for its lifespan.
(Zaarur et al., 2019)	*atgl-1* expression is elevated in *eat-2(ad465)* and required for its Age phenotype (Fig. 2).
(Wang et al., 2020)	Methanol extracts from velvet antler (from Sika deer) increases *eat-2(ad1116)* lifespan under oxidative stress (Fig. 3C).
(Ding et al., 2020)	*eat-2(ad1116)* feeds more slowly than N2, in an assay utilizing bioluminescent bacteria (Fig. 2E,F).
(Chen et al., 2020)	Oenothein B (from *Eucalyptus*) does not further increase *eat-2(ad1116)* lifespan (Fig. 5B).
(Essmann et al., 2020)	*eat-2(ad1116)* does not improve cuticle health or whole-body stiffness during aging, and its lifespan is not further increased by metformin.
(Sun et al., 2020)	Age-related decline in lysosomal function is suppressed in *eat-2(ad1116)*, and lysosome-defective mutants disrupt *eat-2(ad1116)* longevity.
(Lu et al., 2020b)	Tectochrysin (flavonoid compound) does not further increase *eat-2(ad1116)* lifespan (Fig. 5D).
(Ng et al., 2020)	FW1256 (H_2S donor drug) further increases *eat-2(ad1116)* lifespan without additional delays in developmental timing and reduction in fertility.
(Viri et al., 2020)	Compared to N2, *eat-2(ad456)* accumulates more *E. coli* in the posterior intestinal lumen (Fig. 5).

bioRxiv preprint doi: https://doi.org/10.64898/2026.03.11.711062; this version posted July 13, 2026. The copyright holder for this preprint (which was not certified by peer review) is the author/funder, who has granted bioRxiv a license to display the preprint in perpetuity. It is made available under a CC-BY 4.0 International license.

(Lu et al., 2020a)	Secoisolariciresinol diglucoside (a phytoestrogen) does not further increase *eat-2(ad1116)* lifespan (Fig. 6B).
(Farias-Pereira et al., 2020)	Kahweol (diterpene in coffee) does not reduce fat content in *eat-2(ad1116)*, which is reduced compared to N2 (Figure 5A).
(Zhu et al., 2019)	Naringin (a dihydroflavonoid) does not further increase *eat-2(ad1116)* lifespan (Fig 7A).
(Zeng et al., 2021)	Trigonelline does not further increase *eat-2(ad1116)* lifespan (Fig 6D).
(Jiang et al., 2022)	Sonneradon A (extract from *Sonneratia apetala*) increases *eat-2(ad465)* lifespan (Fig. 2F).
(Lin et al., 2022)	*idha-1* mRNA levels (and of other TCA cycle genes) are elevated in *eat-2(ad1116)* (Fig. S2A–E) and *idha-1* RNAi largely abolishes *eat-2(ad1116)* Age (Fig. 4C).
(Campos et al., 2023)	*eat-2(ad1116)* has impaired physical fitness and performance, which worsens with age and is not rescued by long-term exercise, compared to N2.
(Staab et al., 2023)	*eat-2(ad1113)* and *asm-3* have similar lipidome profiles when compared to N2.
(Cho and Park, 2023)	Kahweol (diterpene in coffee) further increases *eat-2(ad1116)* lifespan (Fig. 2).
(Govindhan et al., 2024)	Diosgenin (a steroidal sapogenin) does not further increase *eat-2(ad1116)* lifespan (Fig 3F).
(Cornwell et al., 2024)	Genes involved in amino acid and lipid metabolism, collagen production, and maintenance of muscle mass are downregulated in *eat-2(ad465)*.
(Dawson et al., 2024)	N2, *daf-2(e1370)*, and *eat-2(ad465)* have distinct autophagic flux profiles during postembryonic development; in the last, autophagic flux is modestly elevated throughout (Fig. 5E).
(Mir et al., 2024)	*eat-2(ad465)* is more resistant to heat shock than N2, and this resistance does not depend on *pgrn-1* and *flp* gene expression (Fig. 3).
(Costa et al., 2025)	*eat-2(ad1113)* mutation increases neuronal aggregation of mutant ATXN3 protein in a model of MJD/SCA3, but improves motility.
(Juozaityte et al., 2026)	Memantine (N-methyl-D-aspartate receptor antagonist) induces similar transcriptomic signatures to *eat-2* mutations (Fig. 2E).

Admasu, T.D., Batchu, K.C., Ng, L.F., Cazenave-Gassiot, A., Wenk, M.R. and Gruber, J., 2018. Lipid profiling of *C. elegans* strains administered pro-longevity drugs and drug combinations. Sci Data. 5, 180231.

Artal-Sanz, M. and Tavernarakis, N., 2009. Prohibitin couples diapause signalling to mitochondrial metabolism during ageing in *C. elegans*. Nature. 461, 793-7.

Bansal, A., Zhu, L.J., Yen, K. and Tissenbaum, H.A., 2015. Uncoupling lifespan and healthspan in *Caenorhabditis elegans* longevity mutants. Proc Natl Acad Sci U S A. 112, E277-86.

Campos, J.C., Marchesi Bozi, L.H., Krum, B., Grassmann Bechara, L.R., Ferreira, N.D., Arini, G.S., Albuquerque, R.P., Traa, A., Ogawa, T., van der Bliek, A.M., Beheshti, A., Chouchani, E.T., Van Raamsdonk, J.M., Blackwell, T.K. and Ferreira, J.C.B., 2023. Exercise preserves physical fitness during aging through AMPK and mitochondrial dynamics. Proc Natl Acad Sci U S A. 120, e2204750120.

Carrano, A., Liu, Z., Dillin, A. and Hunter, T., 2009. A conserved ubiquitination pathway determines longevity in response to diet restriction. Nature. 460, 396–399.

Chamoli, M., Singh, A., Malik, Y. and Mukhopadhyay, A., 2014. A novel kinase regulates dietary restriction-mediated longevity in *Caenorhabditis elegans*. Aging Cell. 13, 641–655.

Chen, D., Thomas, E. and Kapahi, P., 2009. HIF-1 modulates dietary restriction-mediated lifespan extension via IRE-1 in *Caenorhabditis elegans*. PLoS Genet. 5, e1000486.

Chen, W., Lin, H.R., Wei, C.M., Luo, X.H., Sun, M.L., Yang, Z.Z., Chen, X.Y. and Wang, H.B., 2018. Echinacoside, a phenylethanoid glycoside from Cistanche

bioRxiv preprint doi: https://doi.org/10.64898/2026.03.11.711062; this version posted July 13, 2026. The copyright holder for this preprint (which was not certified by peer review) is the author/funder, who has granted bioRxiv a license to display the preprint in perpetuity. It is made available under a CC-BY 4.0 International license.

deserticola, extends lifespan of *Caenorhabditis elegans* and protects from Aβ-induced toxicity. Biogerontology. 19, 47-65.

Chen, Y., Onken, B., Chen, H., Zhang, X., Driscoll, M., Cao, Y. and Huang, Q., 2020. Healthy lifespan extension mediated by oenothein B isolated from *Eucalyptus grandis* × *Eucalyptus urophylla* GL9 in *Caenorhabditis elegans*. Food Funct. 11, 2439-2450.

Ching, T.T., Paal, A.B., Mehta, A., Zhong, L. and Hsu, A.L., 2010. *drr-2* encodes an eIF4H that acts downstream of TOR in diet-restriction-induced longevity of *C. elegans*. Aging Cell. 9, 545-57.

Cho, J. and Park, Y., 2023. Kahweol, a coffee diterpene, increases lifespan via insulin/insulin-like growth factor-1 and AMP-activated protein kinase signaling pathways in *Caenorhabditis elegans*. Curr Res Food Sci. 7, 100618.

Cornwell, A.B., Zhang, Y., Thondamal, M., Johnson, D.W., Thakar, J. and Samuelson, A.V., 2024. The *C. elegans* Myc-family of transcription factors coordinate a dynamic adaptive response to dietary restriction. Geroscience. 46, 4827-4854.

Costa, M.D., Da Silva, J.D., Almeida, D., Pereira-Sousa, J., Vilasboas-Campos, D., Fernandes, J.H., Teixeira-Castro, A. and Maciel, P., 2025. Differential effects of lifespan-extending genetic manipulations in an animal model of MJD/SCA3. Mech Ageing Dev. 225, 112064.

Crawford, D., Libina, N. and Kenyon, C., 2007. *Caenorhabditis elegans* integrates food and reproductive signals in lifespan determination. Aging Cell. 6, 715-21.

Curtis, R., O'Connor, G. and DiStefano, P.S., 2006. Aging networks in *Caenorhabditis elegans*: AMP-activated protein kinase (aak-2) links multiple aging and metabolism pathways. Aging Cell. 5, 119-26.

Dawson, Z.D., Sundaramoorthi, H., Regmi, S., Zhang, B., Morrison, S., Fielder, S.M., Zhang, J.R., Hoang, H., Perlmutter, D.H., Luke, C.J., Silverman, G.A. and Pak, S.C., 2024. A fluorescent reporter for rapid assessment of autophagic flux reveals unique autophagy signatures during *C. elegans* post-embryonic development and identifies compounds that modulate autophagy. Autophagy Rep. 3, 2371736.

Ding, S.S., Romenskyy, M., Sarkisyan, K.S. and Brown, A.E.X., 2020. Measuring *Caenorhabditis elegans* spatial foraging and food intake using bioluminescent bacteria. Genetics. 214, 577-587.

Essmann, C.L., Martinez-Martinez, D., Pryor, R., Leung, K.-Y., Krishnan, K.B., Lui, P.P., Greene, N.D.E., Brown, A.E.X., Pawar, V.M., Srinivasan, M.A. and Cabreiro, F., 2020. Mechanical properties measured by atomic force microscopy define health biomarkers in ageing *C. elegans*. Nat Commun. 11, 1043.

Farias-Pereira, R., Park, C.S. and Park, Y., 2020. Kahweol Reduces Food Intake of *Caenorhabditis elegans*. J Agric Food Chem. 68, 9683-9689.

Galbadage, T. and Hartman, P.S., 2008. Repeated temperature fluctuation extends the life span of *Caenorhabditis elegans* in a *daf-16*-dependent fashion. Mech Ageing Dev. 129, 507-14.

Gao, A.W., Smith, R.L., van Weeghel, M., Kamble, R., Janssens, G.E. and Houtkooper, R.H., 2018. Identification of key pathways and metabolic fingerprints of longevity in *C. elegans*. Exp Gerontol. 113, 128-140.

García-Casas, P., Arias-Del-Val, J., Alvarez-Illera, P., Wojnicz, A., de Los Ríos, C., Fonteriz, R.I., Montero, M. and Alvarez, J., 2018. The Neuroprotector Benzothiazepine CGP37157 Extends Lifespan in *C. elegans* Worms. Front Aging Neurosci. 10, 440.

bioRxiv preprint doi: https://doi.org/10.64898/2026.03.11.711062; this version posted July 13, 2026. The copyright holder for this preprint (which was not certified by peer review) is the author/funder, who has granted bioRxiv a license to display the preprint in perpetuity. It is made available under a CC-BY 4.0 International license.

Gelino, S., Chang, J., Kumsta, C., She, X., Davis, A., Nguyen, C., Panowski, S. and Hansen, M., 2016. Intestinal autophagy improves healthspan and longevity in *C. elegans* during dietary restriction. PLoS Genet. 12, e1006135.

Govindhan, T., Amirthalingam, M., Govindan, S., Duraisamy, K., Cho, J.H., Tawata, S., Periyakali, S.B. and Palanisamy, S., 2024. Diosgenin intervention: targeting lipophagy to counter high glucose diet-induced lipid accumulation and lifespan reduction. 3 Biotech. 14, 171.

Greer, E.L. and Brunet, A., 2009. Different dietary restriction regimens extend lifespan by both independent and overlapping genetic pathways in *C. elegans*. Aging Cell. 8, 113-27.

Hahm, J.H., Jeong, C. and Nam, H.G., 2019. Diet restriction-induced healthy aging is mediated through the immune signaling component ZIP-2 in *Caenorhabditis elegans*. Aging Cell. 18, e12982.

Hansen, M., Chandra, A., Mitic, L.L., Onken, B., Driscoll, M. and Kenyon, C., 2008. A role for autophagy in the extension of lifespan by dietary restriction in *C. elegans*. PLoS Genet. 4, e24.

Hansen, M., Taubert, S., Crawford, D., Libina, L., Lee, S.-J. and Kenyon, C., 2007. Lifespan extension by conditions that inhibit translation in *Caenorhabditis elegans.* Aging Cell. 6, 95-110.

Heestand, B., Shen, Y., Liu, W., Magner, D., Storm, N., Meharg, C., Habermann, B. and Antebi, A., 2013. Dietary restriction induced longevity is mediated by nuclear receptor NHR-62 in *Caenorhabditis elegans*. PLOS Genet. 9, e1003651.

Hsu, A., Murphy, C. and Kenyon, C., 2003. Regulation of aging and age-related disease by DAF-16 and heat-shock factor. Science. 300, 1142-1145.

Huang, C., Xiong, C. and Kornfeld, K., 2004. Measurements of age-related changes of physiological processes that predict lifespan of *Caenorhabditis elegans*. Proc Natl Acad Sci U S A. 101, 8084-8089.

Iser, W. and Wolkow, C., 2007. DAF-2/insulin-like signaling in C. elegans modifies effects of dietary restriction and nutrient stress on aging, stress and growth. PLoS One. 2, e1240.

Jia, K. and Levine, B., 2007. Autophagy is required for dietary restriction-mediated life span extension in *C. elegans*. Autophagy. 3, 597–599.

Jiang, S., Jiang, C.P., Cao, P., Liu, Y.H., Gao, C.H. and Yi, X.X., 2022. Sonneradon A Extends Lifespan of *Caenorhabditis elegans* by Modulating Mitochondrial and IIS Signaling Pathways. Mar Drugs. 20.

Juozaityte, V., Pregnolato, C., Abay-Nørgaard, S., Rausch, D.M., McIntyre, R.L., Gerhart-Hines, Z., Pers, T.H., Salcini, A.E. and Clemmensen, C., 2026. The NMDA Receptor Antagonist Memantine Modulates Aging and Stress Resilience. Aging Cell. 25, e70303.

Kim, S.H., Kim, B.K. and Park, S.K., 2018. Selenocysteine mimics the effect of dietary restriction on lifespan via SKN-1 and retards age-associated pathophysiological changes in *Caenorhabditis elegans*. Mol Med Rep. 18, 5389-5398.

Kozlova, A.A., Lotfi, M. and Okkema, P.G., 2019. Cross Talk with the GAR-3 Receptor Contributes to Feeding Defects in *Caenorhabditis elegans eat-2* Mutants. Genetics. 212, 231-243.

Kumar, S., Egan, B., Kocsisova, Z., Schneider, D., Murphy, J., Diwan, A. and Kornfeld, K., 2019. Lifespan extension in *C. elegans* caused by bacterial colonization of the intestine and subsequent activation of an innate immune response. Develop Cell. 49, 100–117.

bioRxiv preprint doi: https://doi.org/10.64898/2026.03.11.711062; this version posted July 13, 2026. The copyright holder for this preprint (which was not certified by peer review) is the author/funder, who has granted bioRxiv a license to display the preprint in perpetuity. It is made available under a CC-BY 4.0 International license.

Lakowski, B. and Hekimi, S., 1998. The genetics of caloric restriction in *Caenorhabditis elegans*. Proc Natl Acad Sci U S A. 95, 13091-13096.

Lee, S.J., Hwang, A.B. and Kenyon, C., 2010. Inhibition of respiration extends *C. elegans* life span via reactive oxygen species that increase HIF-1 activity. Curr Biol. 20, 2131-6.

Lin, Z.H., Chang, S.Y., Shen, W.C., Lin, Y.H., Shen, C.L., Liao, S.B., Liu, Y.C., Chen, C.S., Ching, T.T. and Wang, H.D., 2022. Isocitrate Dehydrogenase Alpha-1 Modulates Lifespan and Oxidative Stress Tolerance in *Caenorhabditis elegans*. Int J Mol Sci. 24, 612.

Lu, M., Tan, L., Zhou, X.G., Yang, Z.L., Zhu, Q., Chen, J.N., Luo, H.R. and Wu, G.S., 2020a. Secoisolariciresinol Diglucoside Delays the Progression of Aging-Related Diseases and Extends the Lifespan of *Caenorhabditis elegans* via DAF-16 and HSF-1. Oxid Med Cell Longev. 2020, 1293935.

Lu, M., Tan, L., Zhou, X.G., Yang, Z.L., Zhu, Q., Chen, J.N., Luo, H.R. and Wu, G.S., 2020b. Tectochrysin increases stress resistance and extends the lifespan of *Caenorhabditis elegans* via FOXO/DAF-16. Biogerontology. 21, 669-682.

Mehta, R., Steinkraus, K., Sutphin, G., Ramos, F., Shamieh, L., Huh, A., Davis, C., Chandler-Brown, D. and Kaeberlein, M., 2009. Proteasomal regulation of the hypoxic response modulates aging in *C. elegans*. Science. 324, 1196–1198.

Meng, F., Li, J., Rao, Y., Wang, W. and Fu, Y., 2018. Gengnianchun Extends the Lifespan of *Caenorhabditis elegans* via the Insulin/IGF-1 Signalling Pathway. Oxid Med Cell Longev. 2018, 4740739.

Mi, X.N., Wang, L.F., Hu, Y., Pan, J.P., Xin, Y.R., Wang, J.H., Geng, H.J., Hu, S.H., Gao, Q. and Luo, H.M., 2018. Methyl 3,4-Dihydroxybenzoate Enhances Resistance to Oxidative Stressors and Lifespan in *C. elegans* Partially via *daf-2/daf-16*. Int J Mol Sci. 19, 1670.

Mir, D.A., Cox, M., Horrocks, J., Ma, Z. and Rogers, A., 2024. Roles of Progranulin and FRamides in Neural Versus Non-Neural Tissues on Dietary Restriction-Related Longevity and Proteostasis in *C. elegans*. J Clin Med Sci. 8, 276.

Murakami, H. and Murakami, S., 2007. Serotonin receptors antagonistically modulate *Caenorhabditis elegans* longevity. Aging Cell. 6, 483-8.

Ng, L.T., Ng, L.F., Tang, R.M.Y., Barardo, D., Halliwell, B., Moore, P.K. and Gruber, J., 2020. Lifespan and healthspan benefits of exogenous H_2S in *C. elegans* are independent from effects downstream of *eat-2* mutation. NPJ Aging Mech Dis. 6, 6.

Onken, B. and Driscoll, M., 2010. Metformin induces a dietary restriction-like state and the oxidative stress response to extend *C. elegans* Healthspan via AMPK, LKB1, and SKN-1. PLoS One. 5, e8758.

Palikaras, K., Lionaki, E. and Tavernarakis, N., 2015. Coordination of mitophagy and mitochondrial biogenesis during ageing in *C. elegans*. Nature. 521, 525-8.

Pan, K.Z., Palter, J.E., Rogers, A.N., Olsen, A., Chen, D., Lithgow, G.J. and Kapahi, P., 2007. Inhibition of mRNA translation extends lifespan in *Caenorhabditis elegans*. Aging Cell. 6, 111-9.

Panowski, S.H., Wolff, S., Aguilaniu, H., Durieux, J. and Dillin, A., 2007. PHA-4/Foxa mediates diet-restriction-induced longevity of *C. elegans*. Nature. 447, 550-5.

Park, S.-K., Link, C. and Johnson, T., 2010. Life-span extension by dietary restriction is mediated by NLP-7 signaling and coelomocyte endocytosis in *C. elegans*. FASEB J. 24, 383–392.

Petrascheck, M., Ye, X. and Buck, L.B., 2007. An antidepressant that extends lifespan in adult *Caenorhabditis elegans*. Nature. 450, 553-6.

bioRxiv preprint doi: https://doi.org/10.64898/2026.03.11.711062; this version posted July 13, 2026. The copyright holder for this preprint (which was not certified by peer review) is the author/funder, who has granted bioRxiv a license to display the preprint in perpetuity. It is made available under a CC-BY 4.0 International license.

Pinkston, J.M., Garigan, D., Hansen, M. and Kenyon, C., 2006. Mutations that increase the life span of *C. elegans* inhibit tumor growth. Science. 313, 971-5.

Powolny, A.A., Singh, S.V., Melov, S., Hubbard, A. and Fisher, A.L., 2011. The garlic constituent diallyl trisulfide increases the lifespan of *C. elegans* via *skn-1* activation. Exp Gerontol. 46, 441-52.

Schlotterer, A., Kukudov, G., Bozorgmehr, F., Hutter, H., Du, X., Oikonomou, D., Ibrahim, Y., Pfisterer, F., Rabbani, N., Thornalley, P., Sayed, A., Fleming, T., Humpert, P., Schwenger, V., Zeier, M., Hamann, A., Stern, D., Brownlee, M., Bierhaus, A., Nawroth, P. and Morcos, M., 2009. *C. elegans* as model for the study of high glucose–mediated life span reduction. Diabetes. 58, 2450–2456.

Shpigel, N., Shemesh, N., Kishner, M. and Ben-Zvi, A., 2019. Dietary restriction and gonadal signaling differentially regulate post-development quality control functions in *Caenorhabditis elegans*. Aging Cell. 18, e12891.

Singh, A., Kumar, N., Matai, L., Jain, V., Garg, A. and Mukhopadhyay, A., 2016. A chromatin modifier integrates insulin/IGF-1 signalling and dietary restriction to regulate longevity. Aging Cell. 15, 694–705.

Staab, T.A., McIntyre, G., Wang, L., Radeny, J., Bettcher, L., Guillen, M., Peck, M.P., Kalil, A.P., Bromley, S.P., Raftery, D. and Chan, J.P., 2023. The lipidomes of *C. elegans* with mutations in asm-3/acid sphingomyelinase and hyl-2/ceramide synthase show distinct lipid profiles during aging. Aging (Albany NY). 15, 650-674.

Sun, Y., Li, M., Zhao, D., Li, X., Yang, C. and Wang, X., 2020. Lysosome activity is modulated by multiple longevity pathways and is important for lifespan extension in *C. elegans*. eLife. 9, e55745.

Syntichaki, P., Troulinaki, K. and Tavernarakis, N., 2007. eIF4E function in somatic cells modulates ageing in *Caenorhabditis elegans*. Nature. 445, 922-6.

Tabrez, S., Sharma, R., Jain, V., Siddiqui, A. and Mukhopadhyay, A., 2017. Differential alternative splicing coupled to nonsense-mediated decay of mRNA ensures dietary restriction-induced longevity. Nat Commun. 8, 306.

Toth, M.L., Sigmond, T., Borsos, E., Barna, J., Erdelyi, P., Takacs-Vellai, K., Orosz, L., Kovacs, A.L., Csikos, G., Sass, M. and Vellai, T., 2008. Longevity pathways converge on autophagy genes to regulate life span in *Caenorhabditis elegans*. Autophagy. 4, 330-8.

Van Raamsdonk, J.M. and Hekimi, S., 2009. Deletion of the mitochondrial superoxide dismutase *sod-2* extends lifespan in *Caenorhabditis elegans*. PLOS Genet. 5, e1000361.

Vilchez, D., Morantte, I., Liu, Z., Douglas, P., Merkwirth, C., Rodrigues, A., Manning, G. and Dillin, A., 2012. RPN-6 determines *C. elegans* longevity under proteotoxic stress conditions. Nature. 489, 263–268.

Viri, V., Cornaglia, M., Atakan, H.B., Lehnert, T. and Gijs, M.A.M., 2020. An in vivo microfluidic study of bacterial transit in *C. elegans* nematodes. Lab Chip. 20, 2696-2708.

Wang, X., Li, H., Liu, Y., Wu, H., Wang, H., Jin, S., Lu, Y., Chang, S., Liu, R., Peng, Y., Guo, Z. and Wang, X., 2020. Velvet antler methanol extracts (MEs) protects against oxidative stress in *Caenorhabditis elegans* by SKN-1. Biomed Pharmacother. 121, 109668.

Wang, Y. and Tissenbaum, H.A., 2006. Overlapping and distinct functions for a *Caenorhabditis elegans* SIR2 and DAF-16/FOXO. Mech Ageing Dev. 127, 48-56.

bioRxiv preprint doi: https://doi.org/10.64898/2026.03.11.711062; this version posted July 13, 2026. The copyright holder for this preprint (which was not certified by peer review) is the author/funder, who has granted bioRxiv a license to display the preprint in perpetuity. It is made available under a CC-BY 4.0 International license.

Yang, Z.Z., Yu, Y.T., Lin, H.R., Liao, D.C., Cui, X.H. and Wang, H.B., 2018. *Lonicera japonica* extends lifespan and healthspan in *Caenorhabditis elegans*. Free Radic Biol Med. 129, 310-322.

Yee, C., Yang, W. and Hekimi, S., 2014. The intrinsic apoptosis pathway mediates the pro-longevity response to mitochondrial ROS in C. elegans. Cell. 157, 897-909.

Yuan, Y., Kadiyala, C.S., Ching, T.T., Hakimi, P., Saha, S., Xu, H., Yuan, C., Mullangi, V., Wang, L., Fivenson, E., Hanson, R.W., Ewing, R., Hsu, A.L., Miyagi, M. and Feng, Z., 2012. Enhanced energy metabolism contributes to the extended life span of calorie-restricted *Caenorhabditis elegans*. J Biol Chem. 287, 31414-26.

Zaarur, N., Desevin, K., Mackenzie, J., Lord, A., Grishok, A. and Kandror, K.V., 2019. ATGL-1 mediates the effect of dietary restriction and the insulin/IGF-1 signaling pathway on longevity in *C. elegans*. Mol Metab. 27, 75-82.

Zeng, W.Y., Tan, L., Han, C., Zheng, Z.Y., Wu, G.S., Luo, H.R. and Li, S.L., 2021. Trigonelline Extends the Lifespan of *C. Elegans* and Delays the Progression of Age-Related Diseases by Activating AMPK, DAF-16, and HSF-1. Oxid Med Cell Longev. 2021, 7656834.

Zhu, B., Jo, K., Yang, P., Tohti, J., Fei, J. and Abudukerim, K., 2019. Aiweixin, a Traditional Uyghur Medicinal Formula, Extends the Lifespan of *Caenorhabditis elegans*. Evid Based Complement Alternat Med. 2019, 3684601.

Supplementary Table 2. Lifespan analysis of Eat mutants on proliferating bacteria (20˚C)

Strain/ condition	Number of deaths/ censored	%P	All deaths			P deaths			p deaths		
			Mean [median] lifespan (days)	% change vs. N2	p vs. N2 (log rank)	Mean [median] lifespan (days)	% change vs. N2	p vs. N2 (log rank)	Mean [median] lifespan (days)	% change vs. N2	p vs. N2 (log rank)
N2	**[C] 429/54**	**45.24**	**16.93 [17]**			**12.82 [13]**			**20.49 [20]**		
	[1] 47/23	34.38	16.79 [18]			11.89 [11]			19.83 [21]		
	[2] 49/4	30.61	17.94 [18]			11.93 [14]			20.59 [18]		
	[3] 56/4	55.36	15.09 [14]			12.19 [12]			18.68 [18]		
	[4] 29/1	44.83	16.83 [18]			13.08 [14]			19.88 [21]		
	[5] 55/5	52.73	16.84 [16]			13.83 [14]			20.19 [20]		
	[6] 57/3	46.55	16.82 [17]			13.22 [13]			20.07 [20]		
	[7] 57/3	45.61	17.46 [17]			13.31 [12]			20.94 [21]		
	[8] 56/4	53.57	17.21 [17]			12.83 [13]			22.27 [24]		
	[9] 23/7	43.48	18.22 [20]			12.20 [13]			22.85 [25]		
eat-1(ad427)	**[C] 153/21**	**28.62**	**17.56 [18]**	**+3.72 [+5.88]**	**0.2246**	**13.33 [12]**	**+3.98 [-7.69]**	**0.2955**	**19.37 [18]**	**-5.47 [-10.00]**	**0.6887**
	[1] 46/14	15.22	17.61 [18]	+4.88 [0]	0.5349	14.57 [14]	+22.54 [+27.27]	0.0358	18.15 [18]	-8.47 [-14.29]	0.4327
	[2] 50/4	18.00	18.82 [18]	+4.91 [0]	0.7106	15.44 [14]	+29.42 [0]	0.0350	19.56 [18]	-5.00 [0]	0.5013
	[3] 57/3	52.63	16.40 [14]	+8.68 [0]	0.1920	12.40 [12]	+1.72 [0]	0.7588	20.85 [20]	+11.62 [+11.11]	0.1242
eat-2(ad1116)	**[C] 362/90**	**13.26**	**22.87 [23]**	**+35.10 [+35.29]**	**<0.0001**	**14.09 [14]**	**+9.91 [+7.69]**	**0.0090**	**24.18 [24]**	**+18.01 [+20.00]**	**<0.0001**
	[1] 55/15	12.73	21.44 [21]	+27.70 [+16.67]	0.0325	15.14 [16]	+27.33 [+45.45]	0.0144	22.35 [21]	+12.71 [0]	0.0297
	[2] 48/9	14.58	20.57 [20]	+14.66 [+11.11]	0.0258	12.50 [14]	+4.78 [0]	0.5505	22.15 [22]	+7.58 [+22.22]	0.1598
	[3] 53/7	11.32	23.00 [22]	+52.42 [+57.14]	<0.0001	12.67 [12]	+3.94 [0]	0.7287	24.32 [25]	+30.19 [+38.89]	<0.0001
	[4] 46/9	13.04	24.57 [25]	+45.99 [+38.89]	<0.0001	16.00 [15]	+22.32 [+7.14]	0.1991	25.85 [25]	+30.03 [+19.05]	<0.0001
	[5] 53/7	9.43	26.06 [28]	+54.75 [+75.00]	<0.0001	17.20 [18]	+24.37 [+28.57]	0.1519	26.98 [28]	+33.63 [+40.00]	<0.0001
	[6] 19/11	20.00	21.79 [23]	+29.55 [+35.29]	<0.0001	10.75 [10]	-18.68 [-23.08]	0.0597	24.73 [23]	+23.22 [+15.00]	0.0007
	[8] 45/15	13.33	22.67 [24]	+31.73 [+41.18]	<0.0001	12.50 [12]	-2.57 [-7.69]	0.9725	24.23 [24]	+8.80 [0]	0.1465
	[9] 43/17	11.63	22.14 [20]	+21.51 [0]	0.0270	16.00 [13]	+31.15 [0]	0.1407	22.95 [20]	+0.44 [-20.00]	0.6186
eat-5(ad464)	**[C] 149/42**	**18.20**	**18.03 [18]**	**+6.50 [+5.88]**	**0.1135**	**12.59 [12]**	**-1.79 [-7.69]**	**0.7401**	**19.24 [18]**	**-6.10 [-10.00]**	**0.0711**
	[1] 48/22	17.02	19.69 [21]	+17.27 [+16.67]	0.4272	12.25 [13]	+3.03 [+18.18]	0.1733	21.18 [21]	+6.81 [0]	0.1944
	[2] 51/10	21.57	17.61 [18]	-1.84 [0]	0.8439	13.82 [14]	+15.84 [0]	0.2287	18.65 [18]	-9.42 [0]	0.1262
	[3] 50/10	16.00	16.88 [16]	+11.86 [+14.29]	0.1080	11.25 [12]	-7.71 [0]	0.5833	17.95 [18]	-3.91 [0]	0.3026
eat-6(ad467)	**[C] 245/203**	**15.21**	**20.44 [21]**	**+20.73 [+23.53]**	**<0.0001**	**16.37 [15]**	**+27.69 [+15.38]**	**<0.0001**	**21.12 [21]**	**+3.07 [+5.00]**	**0.0765**
	[1] 36/25	8.33	21.28 [21]	+26.74 [+16.67]	0.0349	13.67 [12]	+14.97 [+9.09]	0.2667	21.97 [21]	+10.79 [0]	0.0719
	[2] 49/18	8.16	21.14 [21]	+17.84 [+16.67]	0.0255	14.75 [15]	+23.64 [+7.14]	0.2071	21.71 [23]	+5.44 [+27.78]	0.4317
	[3] 34/26	8.82	18.85 [20]	+24.92 [+42.86]	0.0001	14.00 [14]	+14.85 [16.67]	0.0409	19.32 [20]	+3.43 [+11.11]	0.6002
	[4] 35/25	11.43	19.97 [21]	+18.66 [+16.67]	0.0069	18.50 [18]	+41.44 [+28.57]	0.0024	20.16 [21]	+1.41 [0]	0.5797
	[5] 32/28	28.13	20.88 [20]	+23.99 [+25.00]	0.0003	18.89 [18]	+36.59 [+28.57]	0.0073	21.65 [21]	+7.23 [+5.00]	0.1171
	[6] 20/50	21.05	19.90 [23]	+18.31 [+35.29]	0.0002	16.25 [16]	+22.92 [+23.08]	0.0335	20.81 [23]	+3.69 [+15.00]	0.3762
	[7] 39/31	20.51	20.51 [21]	+17.47 [+23.53]	0.0082	15.25 [15]	+14.58 [+25.00]	0.2475	21.87 [21]	+4.44 [0]	0.4528
eat-10(ad606)	**[C] 139/35**	**19.28**	**18.62 [18]**	**+9.98 [+5.88]**	**0.0015**	**12.92 [14]**	**+0.78 [+7.69]**	**0.1945**	**19.93 [20]**	**-2.73 [0]**	**0.9637**
	[1] 44//13	27.27	18.45 [18]	+9.89 [0]	0.8847	12.50 [13]	+5.13 [+18.18]	0.0846	20.69 [21]	+4.34 [0]	0.2752
	[2] 44/13	22.73	17.41 [18]	-2.95 [0]	0.0277	13.60 [14]	+14.00 [0]	0.3379	18.53 [18]	-10.00 [0]	0.1245
	[3] 51/9	7.84	19.80 [20]	+31.21 [+42.86]	<0.0001	12.50 [14]	+2.54 [+16.67]	0.6997	20.43 [20]	+9.37 [+11.11]	0.2451
eat-18(ad1110)	**[C] 138/42**	**39.03**	**17.70 [18]**	**+4.55 [+5.88]**	**0.1529**	**13.24 [13]**	**+3.28 [0]**	**0.2452**	**20.57 [20]**	**+0.39 [0]**	**0.4110**
	[1] 50/20	44.00	16.40 [16]	-2.32 [-11.11]	0.1262	12.55 [13]	+5.55 [+18.18]	0.2330	19.43 [18]	-2.02 [-14.29]	0.6810
	[2] 46/4	32.61	20.04 [21]	+11.71 [+16.67]	0.0277	15.40 [16]	+29.09 [+14.29]	0.0057	22.29 [21]	+8.26 [+16.67]	0.1874
	[3] 42/18	40.48	16.69 [18]	+10.60 [+28.57]	0.0856	12.24 [12]	+0.41 [0]	0.9745	19.72 [20]	+5.57 [+11.11]	0.5404
phm-2(ad597)	**[C] 133/12**	**0**	**26.10 [27]**	**+54.16 [+58.82]**	**<0.0001**				**26.10 [27]**	**+27.38 [+35.00]**	**<0.0001**
	[1] 60/3	0	27.07 [27]	+61.23 [+50.00]	<0.0001				27.07 [27]	+36.51 [+28.57]	<0.0001
	[2] 46/6	0	24.50 [24]	+36.57 [+33.33]	<0.0001				24.50 [24]	+19.00 [+33.33]	0.0011
	[3] 27/3	0	26.67 [25]	+76.74 [+78.57]	<0.0001				26.67 [25]	+42.77 [+38.89]	<0.0001

phm-3(ad493)	**[C] 84/73**	**3.23**	**19.51 [20]**	**+15.24 [+17.65]**	**0.0001**	**15.33 [16]**	**+19.58 [+23.08]**	**0.0098**	**19.67 [20]**	**-4.00 [0]**	**0.8943**
	[1] 32/19	0	20.38 [20]	+21.38 [+11.11]	0.1555				20.38 [20]	+2.77 [-4.76]	0.3388
	[2] 21/25	0	17.29 [18]	-3.62 [0]	0.9233				17.29 [18]	-16.03 [0]	0.0848
	[3] 31/29	9.68	20.13 [18]	+33.40 [+28.57]	<0.0001	15.33 [16]	+25.76 [+33.33]	0.0012	20.64 [20]	+58.67 [+11.11]	0.1913

bioRxiv preprint doi: https://doi.org/10.64898/2026.03.11.711062; this version posted July 13, 2026. The copyright holder for this preprint (which was not certified by peer review) is the author/funder, who has granted bioRxiv a license to display the preprint in perpetuity. It is made available under a CC-BY 4.0 International license.

Supplementary Table 3. Lifespan analysis of Eat mutants on non-proliferating bacteria (20˚C)

Strain/ condition	Number of deaths/censored	Mean [median] lifespan (days)	% change vs. N2	p vs. N2 (log rank)
N2	**[C] 199/9**	**24.69 [24]**		
	[1] 44/1	23.80 [24]		
	[2] 53/4	23.26 [23]		
	[3] 45/1	24.84 [26]		
	[4] 57/3	26.58 [26]		
eat-1(ad427)	**[C] 137/12**	**23.69 [24]**	**-4.05 [0]**	**0.0518**
	[1] 51/1	23.75 [23]	-0.21 [-4.17]	0.7648
	[3] 29/8	25.69 [26]	+3.42 [0]	0.6275
	[4] 57/3	22.61 [24]	-14.94 [-7.69]	0.0029
eat-2(ad1116)	**[C] 192/15**	**27.40 [28]**	**+10.98 [+16.67]**	**0.0078**
	[1] 45/2	26.62 [26]	+11.85 [+8.33]	0.0620
	[2] 25/3	26.68 [26]	+14.70 [+13.04]	0.1815
	[3] 63/9	28.22 [28]	+13.61 [+7.69]	0.0115
	[4] 59/1	27.42 [28]	+3.16 [+7.69]	0.8892
eat-5(ad464)	**[C] 190/31**	**23.34 [23]**	**-5.47 [-4.17]**	**0.0816**
	[1] 49/4	21.04 [18]	-11.60 [-25.00]	0.1128
	[2] 52/3	19.90 [19]	-14.45 [-17.39]	0.0050
	[3] 44/9	27.14 [28]	+9.26 [+7.69]	0.1321
	[4] 45/15	26.11 [26]	-1.77 [0]	0.7414
eat-6(ad467)	**[C] 196/40**	**23.38 [23]**	**-5.31 [-4.17]**	**0.0221**
	[1] 47/1	21.00 [18]	-11.76 [-25.00]	0.0958
	[2] 57/4	22.44 [21]	-3.53 [-8.70]	0.6250
	[3] 57/10	26.70 [26]	+7.49 [0]	0.3194
	[4] 35/25	22.71 [21]	-14.56 [-19.23]	0.0020
eat-10(ad606)	**[C] 162/40**	**21.46 [22]**	**-13.08 [-8.33]**	**<0.0001**
	[1] 56/1	20.63 [22]	-13.32 [-8.33]	0.1648
	[2] 46/3	20.20 [19]	-13.16 [-17.39]	0.0135
	[3] 28/8	24.54 [26]	-1.21 [0]	0.5546
	[4] 32/28	22.06 [21]	-17.01 [-19.23]	0.0082
eat-18(ad1110)	**[C] 203/39**	**24.48 [24]**	**-0.85 [0]**	**0.5378**
	[1] 32/1	25.41 [25]	+6.76 [+4.17]	0.6891
	[2] 47/5	19.77 [19]	-15.00 [-17.39]	0.0019
	[3] 82/15	27.40 [28]	+10.30 [+7.69]	0.0441
	[4] 42/18	23.36 [24]	-12.11 [-7.69]	0.0065
phm-2(ad597)	**[C] 227/11**	**28.94 [28]**	**+17.21 [+16.67]**	**<0.0001**
	[1] 54/1	29.83 [30]	+25.34 [+25.00]	<0.0001
	[2] 47/3	28.53 [30]	+22.66 [+30.43]	0.0056
	[3] 70/3	30.29 [30]	+21.94 [+15.38]	<0.0001
	[4] 56/4	26.75 [28]	+0.64 [+7.69]	0.5769
phm-3(ad493)	**[C] 174/53**	**23.44 [23]**	**-5.06 [-4.17]**	**0.0247**
	[1] 43/1	22.67 [23]	-4.75 [-4.17]	0.0694
	[2] 51/1	22.10 [21]	-4.99 [-8.70]	0.2067
	[3] 54/17	23.65 [23]	-4.79 [-11.54]	0.2447
	[4] 26/34	26.88 [26]	+1.13 [0]	0.7480

Supplementary Table 4. Lifespan analysis of 3 *eat-2* mutants on proliferating bacteria (20˚C)

Strain/ condition	Number of deaths/ censored	%P	All deaths			P deaths			p deaths		
			**Mean [median] lifespan (days)**	**% change vs. N2**	**p vs. N2 (log rank)**	**Mean [median] lifespan (days)**	**% change vs. N2**	**p vs. N2 (log rank)**	**Mean [median] lifespan (days)**	**% change vs. N2**	**p vs. N2 (log rank)**
N2	**[C] 111/9**	**51.32**	**16.71 [17]**			**12.82 [14]**			**20.81 [22]**		
	[1] 57/3	52.63	16.46 [14]			12.53 [12]			20.81 [21]		
	[2] 54/6	50.00	16.98 [17]			13.15 [14]			20.81 [22]		
eat-2(ad465)	**[C] 74/41**	**7.96**	**20.19 [19]**	**+20.83 [+11.76]**	**<0.0001**	**13.83 [14]**	**+7.88 [0]**	**0.0322**	**20.75 [21]**	**-0.29 [-4.55]**	**0.3722**
	[1] 47/33	8.51	21.13 [21]	+28.37 [+50.00]	<0.0001	13.75 [13]	+9.74 [+8.33]	0.0142	21.81 [21]	+4.81 [0]	0.2125
	[2] 27/8	7.41	18.56 [17]	+9.31 [0]	0.1626	14.00 [14]	+6.46 [0]	0.2465	18.92 [18]	-9.08 [-18.18]	0.2819
eat-2(ad1113)	**[C] 102/48**	**9.07**	**20.58 [22]**	**+23.16 [+29.41]**	**<0.0001**	**14.33 [14]**	**+11.78 [0]**	**0.0125**	**21.18 [22]**	**+1.78 [0]**	**0.1844**
	[1] 57/23	7.02	21.21 [21]	+28.86 [+50.00]	0.0019	15.50 [15.5]	+23.70 [+29.17]	0.0021	21.64 [21]	+3.99 [0]	0.2948
	[2] 45/25	11.11	19.78 [22]	+9.31 [+29.41]	0.0258	13.40 [14]	+1.90 [0]	0.7627	20.58 [22]	-1.11 [0]	0.5340
eat-2(ad1116)	**[C] 113/17**	**9.83**	**22.53 [23]**	**+34.83 [+35.29]**	**<0.0001**	**13.09 [14]**	**+2.11 [0]**	**0.7851**	**23.55 [24]**	**+13.17 [+9.09]**	**0.0002**
	[1] 53/7	11.32	23.74 [24]	+44.23 [+71.43]	<0.0001	12.83 [13]	+2.39 [+8.33]	0.8930	25.13 [24]	+20.76 [+14.29]	0.0008
	[2] 60/10	8.33	21.47 [22]	+26.44 [29.41]	<0.0001	13.40 [14]	+1.90 [0]	0.7909	22.20 [22]	+6.68 [0]	0.0490

bioRxiv preprint doi: https://doi.org/10.64898/2026.03.11.711062; this version posted July 13, 2026. The copyright holder for this preprint (which was not certified by peer review) is the author/funder, who has granted bioRxiv a license to display the preprint in perpetuity. It is made available under a CC-BY 4.0 International license.

Supplementary Table 5. Lifespan analysis of *eat-2* and *phm-2* mutants on small and large bacterial lawns (20˚C)

Strain/ condition	Number of deaths/censored	Mean [median] lifespan (days)	% change vs. N2	p vs. N2 (log rank)	% change vs. Small lawn	p vs. Small lawn (log rank)
N2<br>Small lawn	**[C] 163/17**<br>[1] 47/13<br>[2] 29/1<br>[3] 59/1<br>[4] 28/2	**17.24 [17]**<br>17.47 [17]<br>17.10 [19]<br>16.71 [16]<br>18.11 [18]				
N2<br>Large lawn	**[C] 188/22**<br>[1] 47/13<br>[2] 55/5<br>[3] 56/4<br>[4] 30/0	**14.98 [14]**<br>15.70 [15]<br>16.29 [15]<br>13.46 [12]<br>14.27 [12]			**-13.11 [-17.65]**<br>-10.13 [-11.76]<br>-4.74 [-21.05]<br>-19.45 [-25.00]<br>-21.20 [-33.33]	**0.0054**<br>0.0262<br>0.9742<br>0.0110<br>0.0391
eat-2(ad1116)<br>Small lawn	**[C] 130/50**<br>[1] 17/13<br>[2] 44/16<br>[3] 43/17<br>[4] 26/4	**18.74 [19]**<br>18.88 [20]<br>18.05 [19]<br>18.98 [20]<br>19.42 [19]	**+8.70 [+11.76]**<br>+8.07 [+17.65]<br>+5.56 [0]<br>+13.58 [+25.00]<br>+7.23 [+5.56]	**0.0013**<br>0.0391<br>0.2488<br>0.0183<br>0.2974		
eat-2(ad1116)<br>Large lawn	**[C] 162/73**<br>[1] 29/26<br>[2] 35/25<br>[3] 50/10<br>[4] 48/12	**20.94 [21]**<br>20.03 [22]<br>20.31 [19]<br>20.82 [20]<br>22.08 [24]	**+39.79 [+50.00]**<br>+27.58 [+46.67]<br>+24.68 [+26.67]<br>+54.68 [+66.67]<br>+54.73 [+100.00]	**<0.0001**<br><0.0001<br>0.0012<br><0.0001<br><0.0001	**+11.74 [+10.53]**<br>+6.09 [+10.00]<br>+12.52 [0]<br>+9.69 [0]<br>+13.70 [+26.32]	**0.0016**<br>0.8632<br>0.0807<br>0.1057<br>0.1435
phm-2(ad597)<br>Small lawn	**[C] 107/43**<br>[1] 36/24<br>[2] 23/7<br>[4] 48/12	**23.90 [24]**<br>20.92 [22]<br>25.00 [25]<br>25.60 [26]	**+38.63 [+41.18]**<br>+19.75 [+29.41]<br>+46.20 [+31.58]<br>+41.36 [+44.44]	**<0.0001**<br><0.0001<br><0.0001<br><0.0001		
phm-2(ad597)<br>Large lawn	**[C] 94/146**<br>[1] 31/29<br>[2] 8/22<br>[3] 36/54<br>[4] 19/41	**22.32 [23]**<br>21.00 [22]<br>24.50 [23]<br>22.31 [23]<br>23.58 [24]	**+49.00 [+64.29]**<br>+33.76 [+46.67]<br>+50.40 [+53.33]<br>+65.75 [+91.67]<br>+65.24 [+100.00]	**<0.0001**<br><0.0001<br>0.0010<br><0.0001<br><0.0001	**-6.61 [-4.17]**<br>+0.38 [0]<br>-2.00 [-8.00]<br><br>-7.89 [-7.69]	**0.0051**<br>0.9866<br>0.8733<br><br>0.0278
